# Emerging IL-12 family cytokines in the fight against fungal infections

**DOI:** 10.1016/j.cyto.2018.05.019

**Published:** 2018-11

**Authors:** Aiysha Thompson, Selinda J. Orr

**Affiliations:** Division of Infection and Immunity, Cardiff University School of Medicine, Cardiff CF14 4XN, Wales, United Kingdom

**Keywords:** Fungal, IL-12, IL-23, IL-27, IL-35

## Abstract

Invasive fungal infections cause approximately 1.5 million deaths per year worldwide and are a growing threat to human health. Current anti-fungal therapies are often insufficient, therefore studies into host-pathogen interactions are critical for the development of novel therapies to improve mortality rates. Myeloid cells, such as macrophages and dendritic cells, express pattern recognition receptor (PRRs), which are important for fungal recognition. Engagement of these PRRs by fungal pathogens induces multiple cytokines, which in turn activate T effector responses. Interleukin (IL)-12 family members (IL-12p70, IL-23, IL-27 and IL-35) link innate immunity with the development of adaptive immunity and are also important for regulating T cell responses. IL-12 and IL-23 have established roles during anti-fungal immunity, whereas emerging roles for IL-27 and IL-35 have recently been reported. Here, we discuss the IL-12 family, focusing on IL-27 and IL-35 during anti-fungal immune responses to pathogens such as *Candida* and *Aspergillus*.

## Highlights

• IL-12 and IL-23 have established roles during anti-fungal immunity.
• IL-27 promotes regulatory effector responses during fungal infections.
• IL-35 drives T cell differentiation to produce anti-inflammatory responses.
• Increasing evidence for IL-12 family cytokines in maintaining anti-fungal immune homeostasis.

## 1 Introduction

Interleukin (IL)-12 family members (IL-12p70, IL-23, IL-27 and IL-35) link innate immunity with the development of adaptive immunity [1]. They are important in regulating T cell responses and while structurally similar, they display different functional activities. IL-12 and IL-23 are both considered to be pro-inflammatory [2], [3]. IL-12 predominantly favors T helper (Th)1 cell differentiation [4], [5], [6], whereas IL-23 enhances Th17 responses and is involved in the activation and expansion of memory T-cells [7], [8]. IL-27 has both pro- and anti-inflammatory properties and is a potent T cell immunomodulator. IL-27 was originally reported to enhance Th1 cell differentiation [9], [10]. However, subsequent studies highlight more nuanced roles linked with suppression of adaptive immunity, where IL-27 inhibits IL-2 signaling, antagonizes Th17 cells and promotes regulatory T cells (Tregs) responses [11], [12], [13], [14], [15], [16]. Lastly, IL-35 induces Tregs and regulatory B cells (Bregs) and suppresses T cell responses [17]. IL-12 and IL-23 have established roles during anti-fungal immunity [7], [18], [19], [20], [21], [22], [23], [24], [25], [26], [27], [28], [29] and new roles for IL-27 and IL-35 have recently been reported [20], [23], [30]. Importantly, the new roles reported for IL-27 as a regulatory cytokine emerged from infection studies suggesting that IL-27 may also have important roles in fungal control. This review will focus on the role of these newer family members during fungal infections, such as *Candida* and *Aspergillus* infections.

*Candida* species (spp). cause various types of disease, from mucosal infections (eg. oral and vaginal thrush) to life threatening invasive candidiasis. *Candida* spp. bind various Pattern Recognition Receptors (PRRs) such as C-type lectin-like receptors (CLRs) including Dectin-1, Toll-like receptors (TLRs) and Nucleotide-binding Oligomerisation domain-like receptors (NLRs) on myeloid cells [31]. This induces multiple cytokines, which in turn activate Th1 and Th17 responses [32], [33], [34], [35], [36], [37], [38]. The production of IFN-γ by Th1 cells has been shown to be crucial in the control of systemic candidiasis in a murine model [21], [39], [40], [41]. Whereas, the Th17 response protects against oropharyngeal and mucocutaneous models of candidiasis while increasing disease susceptibility in a gastrointestinal model [7], [29], [42], [43].

*Aspergillus fumigatus* is an airborne fungus that causes various types of disease including invasive pulmonary aspergillosis (IPA) and allergic bronchopulmonary aspergillosis (ABPA). An important role of the host immune response is to limit spore germination and restrict hyphal invasion. Spore swelling, the first step of germination, exposes fungal ligands that bind to CLRs and TLRs resulting in inflammatory cytokine production [44], [45]. This then induces a Th1 response and a much smaller Th17 response in a pulmonary infection model [25], [46] whereas a Th2 response is induced in an allergic model of disease [47], [48].

The T cell responses mediated by these different pathogens (*Candida* and *Aspergillus*) are likely controlled by the ligands exposed on their cell surface and by the environment (e.g. systemic, mucosal, pulmonary) of the infection. The yeast form of *C. albicans* expresses mannans on the cell surface and β-glucans are rapidly exposed at bud scars whereas dormant *Aspergillus* conidia are covered in a rodlet layer that initially hides β-glucans from the immune system [44], [49], [50]. Mannans present on the yeast form of *C. albicans* have been shown to induce IL-1β and IL-23 in a Dectin-2 dependent manner [37]. Additionally, both yeast and hyphal forms of *C. albicans* induced Dectin-2-dependent Th17 differentiation *in vitro* and *Il17a* deficient mice were more susceptible to systemic infection with *C. albicans* [37]. Further to this, dendritic cells (DCs) activated by Dectin-1 engagement by curdlan, a β-glucan preparation, demonstrated Th17 cell differentiation of CD4^+^ T cells *in vitro*, and the use of curdlan as an adjuvant *in vivo*, promoted both Th17 and Th1 responses [34]. We have previously demonstrated differences in T cell differentiation *in vitro* and *in vivo*. Heat killed yeast predominantly induced Th17 differentiation *in vitro*, whereas, mice systemically infected with *C. albicans* displayed a Th1-biased response *in vivo* [19], therefore the environment is also important for determining T cell bias. In the context of *A. fumigatus* infections, Th1 and Th17 CD4^+^ T cells are driven via TLR/MyD88 and Dectin-1 respectively. TLR2 and TLR4 recognise *A. fumigatus* conidia [51], whereas Dectin-1 recognises later morphological forms such as swollen conidia and early germlings [44]. It is possible that the TLRs initiate a Th1 response to *A. fumigatus* and then Dectin-1 induces the Th17 response once germination of *A. fumigatus* is further underway. As *C. albicans* and *A. fumigatus* engage different combinations of PRRs, interactions between these PRRs likely dictate the balance between Th1 and Th17 responses to these two pathogens.

## 2 IL-12 family cytokines

### 2.1 Structure

**Fig. 1:**
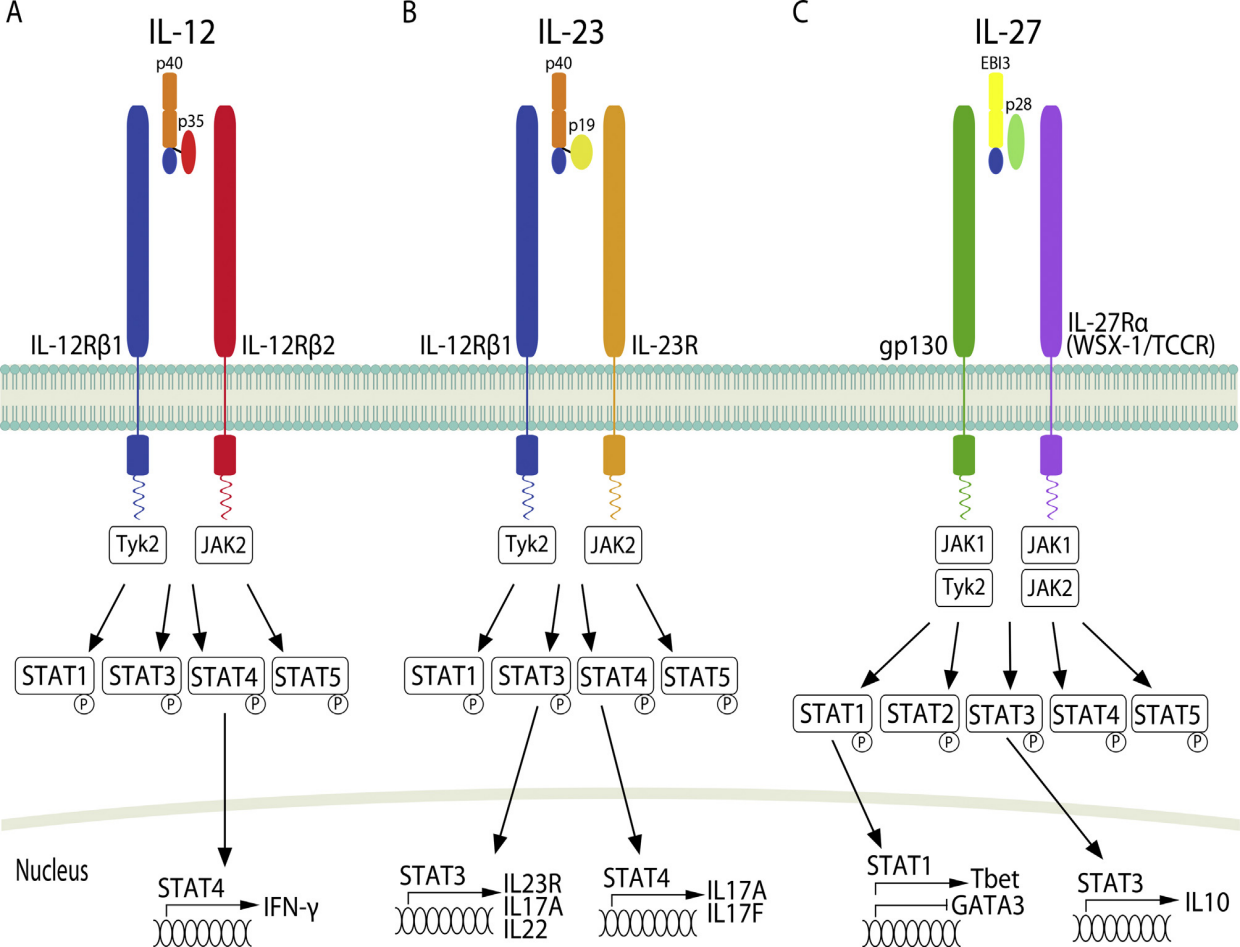
IL-12, IL-23 and IL-27 cytokines and their receptors. (A) IL-12 is a heterodimeric cytokine made up of a light chain IL-12p35 and a heavy chain IL-12p40. The IL-12 receptor is made up of IL-12Rβ1 and IL-12Rβ2 and signals through Tyk2 and JAK2 to activate STAT1, 3, 4 and 5. STAT4 induces nuclear translocation of IFN-γ. (B) The IL-12p40 component of IL-23 can dimerise with IL-23p19 to form IL-23. The IL-23 receptor is comprised of IL-12Rβ1 and IL-23R and signals through Tyk2 and JAK2 to activate STAT1, 3, 4 and 5. STAT3 induces nuclear translocation of the IL-23R, IL-17A and IL-22; and STAT4 induces nuclear translocation of IL-17A/F. (C) IL-27 is composed of EBI3 and IL-27p28. IL-27 binds a receptor composed of gp130 and IL-27Rα and signals through JAK1, Tyk2 and JAK2 to activate STAT1, 2, 3, 4 and 5. STAT1 induces nuclear translocation of Tbet and inhibition of GATA3; and STAT3 induces nuclear translocation of IL-10.

**Fig. 2:**
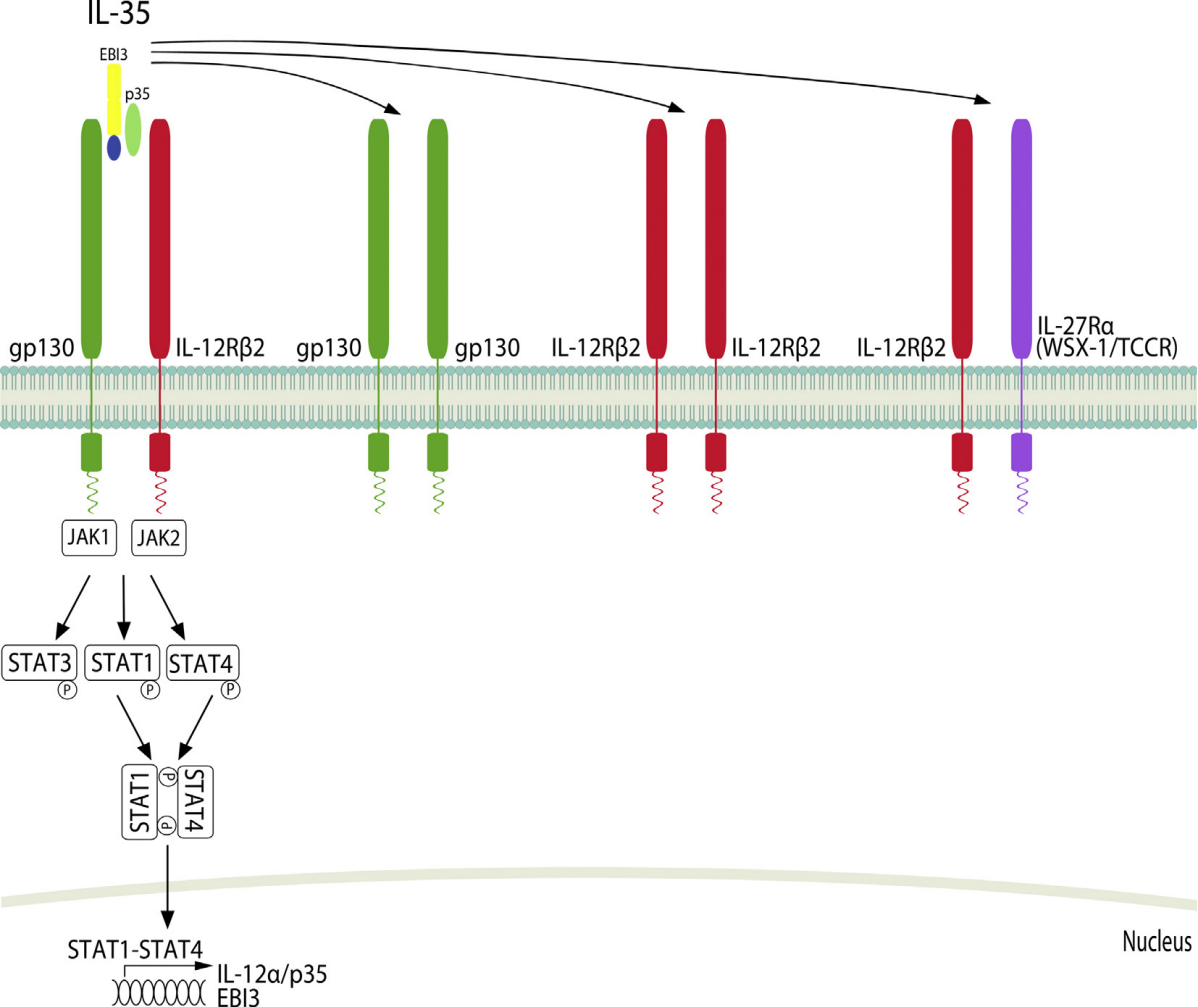
IL-35 cytokines and their receptors. IL-35 is a heterodimeric cytokine made up of EBI3 (shared with IL-27) and IL-12p35 (shared with IL-12). gp130 couples with IL-12Rβ2 to form the IL-35 receptor complex and signals through JAK1 and JAK2 to activate STAT1, 3 and 4. STAT-1-STAT4 form a heterodimer to induce nuclear translocation and induction of IL-12α/p35 and EBI3. Additionally, homodimers of gp130 or IL-12Rβ2 have been shown to elicit a partial IL-35 induced response in T cells [86], and IL-12Rβ2 can also couple with IL-27Rα to also form an IL-35 receptor complex in B cells [17].

IL-12 family cytokines consist of four heterodimeric proteins (Fig. 1, Fig. 2), which are mainly produced by myeloid cells. This has been extensively reviewed elsewhere [22], [52], [53], [54], [55], [56], [57]. IL-12 is made up of 2 subunits, IL-12p35 and IL-12p40, which together form active IL-12p70. IL-12p70 is a very potent activator of Th1 cell responses [4], [5]. IL-12p40 is also expressed and secreted as a monomer and a homodimer [58], [59]. The p40 monomer has no biological function whereas the homodimer is an antagonist of IL-12p70 by competitively binding the IL-12R [60]. The IL-12p40 homodimer enhances alloantigen-specific Th1 development suggesting similar functional activity to IL-12p70 under certain conditions [61]. IL-23 is composed of IL-23p19 and IL-12p40 (shared with IL-12p70). IL-23 is tightly regulated and is functionally distinct from IL-12. IL-27 is a heterodimer made up of IL-27p28 and Epstein Barr virus induced gene 3 (EBI3) subunits [10], [62]. IL-27p28 has also been shown to be expressed as a monomer, which can inhibit IL-6, IL-27 and IL-11 signalling through the common gp130 signalling receptor [63], [64]. IL-27 modulates pro- and anti-inflammatory signaling [65], [66], [67]. IL-35 consists of the EBI3 subunit (shared with IL-27) and the IL-12p35 subunit (shared with IL-12) [68]. IL-35 promotes Treg responses and suppresses Th1 and Th17 responses. Treg cells deficient in either *Ebi3* or *Il12a* (IL-12p35) display a reduced ability to inhibit effector T cell proliferation [69], [70], [71], [72].

### 2.2 Signaling

IL-12 binds to IL-12Rβ1 and IL-12Rβ2 and signals via Janus associated kinase 2 (JAK2) and tyrosine kinase 2 (Tyk2) to activate various signal transducer and activator of transcription (STAT) family members [3], [73] (Fig. 1). Patients with defective IL12B or IL12RB1 [74] and cells from mice deficient for Tyk2 or STAT4 showed reduced IFN-γ production in T cells and natural killer (NK) cells [75], [76], [77]. IL-23 binds IL-12Rβ1 and IL-23R [78], [79] and signals in a similar manner to IL-12 [79], [80] (Fig. 1). JAK2 and Tyk2 constitutively associate with IL-23R and IL-12R-β1 chains respectively [79]. IL-23 activated STAT3 and STAT4 is critical for IL-17 induction from Th17 cells [81], [82]. The IL-23R and IL-12R-β1 chains alone lack intrinsic enzymatic activity [2], [3], [79], [83].

IL-27 signals through IL-27Rα (WSX-1/TCCR) paired with glycoprotein (gp)130 [84] (Fig. 1). IL-27 can bind WSX-1 with low affinity in the absence of gp130, however both IL-27R subunits are required for effective signal transduction [10], [84]. IL-27 signaling activates various JAK and STAT family members in a cell-specific manner. For example, IL-27 activates both STAT1 and STAT3 in naïve CD4^+^ T cells but in fully active T cells only STAT3 activation is retained [85]. IL-35 signaling is unconventional as it utilizes different receptor combinations including IL-12Rβ2 and gp130 homodimers and heterodimers (Fig. 2). STAT1 and/or STAT4 are activated depending on which receptor homodimer/heterodimer is engaged. In B cells IL-35 signals through IL-12Rβ2 and WSX-1 to activate STAT1 and STAT4. More work is required to fully understand IL-35 signaling downstream of the different homodimers or heterodimers [17], [86].

## 3 IL-12 family cytokines in anti-fungal host defense

### 3.1 IL-12

IL-12 is induced by microbial products in monocytes, macrophages and DCs and acts on NK and T cells to induce IFN-γ. IFN-γ in turn activates monocytes and macrophages to induce further IL-12 production [87]. This positive feedback mechanism helps protect against certain pathogens that induce low IL-12 levels [88]. Deficiency of IL-12 or IL-12R genes leads to impaired cell mediated immunity and enhanced disease susceptibility [89], [90], [91], [92]. We will only briefly discuss the role of IL-12 during *Candida* infections as this has been reviewed elsewhere [22], [93].

Several *C. albicans* infection experiments have been performed in mice deficient in different components of the IL-12 family (Table 1). However, due to subunit sharing between different family members, interpretation of these results is rather complex. Overall, IL-12 appears to be important during systemic infection but less important during mucocutaneous infections. *Il12p40^-/-^* mice were susceptible to oral infection with *C. albicans*, however, this was mainly attributed to IL-23 and Th17 responses [29]. Interestingly, *Il12p35^-/-^* mice demonstrated disseminated infection following oral infection with *C. albicans* suggesting that while IL-12 is not overly important in the local response during an oral infection, it controls systemic dissemination [29]. In addition, *Il12p35^-/-^* and *IFN*-γ*^-/-^* mice displayed enhanced susceptibility to systemic infection with *C. albicans* [7], [39], [40], [41], indicating an important role for IL-12/Th1 responses during systemic infection. *Il12p40^-/-^* mice displayed variable susceptibility to systemic infection in different studies [7], [26], [94]. Additionally, in a mouse model where mannoproteins from the fungal pathogen *Cryptococcus neoformans* were shown to protect against systemic *C. albicans* infection, this protective response was reversed upon IL-12 blockade [95]. Further, when IL-12 was fused to enolase (a *C. albicans* antigen) and administered during systemic infection with *C. albicans*, mice demonstrated increased survival and decreased fungal burden in their kidneys [27]. However, administration of IL-12 during systemic infection increased disease severity due to IFN-γ mediated pathology [96]. These data suggest that while IL-12 and IFN-γ are important for controlling systemic infections with *C. albicans*, this response needs to be finely tuned for optimal fungal clearance while minimising pathology.

**Table 1:** Effects of different *Candida* and *Aspergillus* infections on *Il12p40^-/-^*, *Il12p35^-/-^*, *Il23p19^-/-^* and *Il27ra^-/-^* mice.

Genotype	Pathogen	Outcome	References
*Il12p40^-/-^*	*C. albicans*	Susceptible to oral challenge	Farah et al. [26]
	*C. albicans*	Reduced fungal burden during systemic challenge (1, 3 days post infection) but increased fungal burden following re-infection	Mencacci et al. [94]
	*C. albicans*	No effect during systemic challenge	Netea et al. 2003 [170]
	*C. albicans*	No effect during systemic challenge (5 days post infection)	Farah et al. 2006 [26]
	*C. albicans*	Increased fungal burden during gastrointestinal challenge (3, 10 days post infection)	Zelante et al. [7]
	*C. albicans*	Susceptible to cutaneous challenge	Kagami et al. [112]
	*C. albicans*	Increased fungal burden during gastrointestinal challenge	Mencacci et al. [94]
	*A. fumigatus*	Increased fungal burden during IPA in immunosuppressed mice	Cenci et al. [99]
	*A. fumigatus*	Reduced fungal burden during IPA	Zelante et al. [7]

*Il12p35^-/-^*	*C. albicans*	Systemic dissemination during oral challenge	Conti et al. [29]
	*C. albicans*	Susceptible to gastrointestinal challenge	Zelante et al. [7]
	*C. albicans*	No effect during cutaneous challenge	Kagami et al. [112]
	*A. fumigatus*	Reduced fungal burden during IPA	Zelante et al. [7]

*Il23p19^-/-^*	*C. albicans*	Susceptible to oral challenge	Conti et al. [29]
	*C. albicans*	No effect during gastrointestinal challenge	Zelante et al. [7]
	*C. albicans*	No effect during vulvovaginal challenge	Yano et al. [111]
	*C. albicans*	Susceptible to cutaneous challenge	Kagami et al. [112]
	*A. fumigatus*	Reduced fungal burden during IPA	Zelante et al. [7]

*Il27ra^-/-^*	*C. albicans*	No significant effect during systemic challenge	Patin et al. [30]
	*C. parapsilosis*	Enhanced fungal clearance and reduced fungal burden in the kidneys during systemic challenge (6 weeks post infection)	Patin et al. [30]

The importance of IL-12 has also been demonstrated during *A. fumigatus* infections. Human DCs infected with *A. fumigatus* induced IL-12p70 production and promoted IFN-γ production from CD4^+^ T cells [20]. Rivera et al. [25] showed that Th1 and Th17 responses are induced during an *in vivo* model of *A. fumigatus* lung infection. The authors showed that TLR and Dectin-1 pathways modulate the balance between Th1 and Th17 populations by controlling production of IL-12 family members. In addition, IL-12 has been shown to induce human monocytes to damage *A. fumigatus* hyphae via an oxidative burst response in an IFN-γ-independent manner [97].

During an *in vivo* infection model with *A. fumigatus*, Cenci et al. [98] compared the immune response of resistant (intact DBA/2 mice) and susceptible (leukopenic DBA/2 mice) mice. Interestingly, the authors demonstrated that resistant mice produced high levels of IL-12, TNF and IFN-γ while susceptible mice produced an IL-10 and IL-4 skewed response. In addition, immunosuppressed *Il12p40^-/-^* and *Ifng^-/-^* mice were more susceptible to invasive pulmonary aspergillosis [99]. In agreement with this, Nagai et al. [100] showed that administration of IFN-γ protected mice from invasive aspergillosis. Furthermore, Delsing et al. [101] showed that adjunctive therapy with IFN-γ and an antifungal drug partially restored immune function in patients with invasive *Candida* and/or *Aspergillus* infections indicating that further clinical studies are warranted to fully determine the potential clinical benefits of IFN-γ therapy during invasive fungal infections.

### 3.2 IL-23

IL-23 is involved in the maintenance of Th17 cells and the activation of memory T-cells [78], [102]. It induces CD4^+^ Th17 cells to produce IL-17 and IL-22 [80], [103], [104], [105]. IL-17A is a proinflammatory cytokine that promotes neutrophil recruitment via upregulation of other cytokines and chemokines [106]. While the IL-23-IL-17 pathway is important for protective responses against infection, it can also contribute to autoimmunity and increased pathology [107], [108]. IL-23 is also important for regulation of innate lymphocyte cells (e.g. innate lymphoid, NK, NKT and γδ T-cells [109]).

*C. albicans* induces IL-23p19 and/or IL-23 in cultured macrophages and DCs and at sites of inflammation during infection [19], [24]. Interestingly, human monocyte derived DCs produced IL-23 when challenged with *C. albicans* hyphae, whereas *C. albicans* yeast induced IL-12 production and IL-23 was only produced with high concentrations of yeast [110]. This suggests that the hyphal form of *C. albicans* triggers the Th17 response *in vivo*.

During systemic infection with *C. albicans,* IL-23 was dispensable, however the IL-23/Th17 pathway is important during chronic muccocutaneous candidiasis (CMC) [7]. *Il23p19^-/-^*, and *Il17ra^-/-^* mice were more susceptible to oral *C. albicans* infection [29]. However, Yano et al. (2012) found that fungal burden and S100A8/S100A9 alarmin-mediated neutrophil recruitment was normal in *Il23p19^-/-^*, *Il17ra^-/-^* and *Il22^-/-^* mice in a mouse vaginal infection model suggesting that the vaginal alarmin S100 response is independent of the IL-23/Th17 axis [111]. In contrast, several other studies have found important roles for Th17 cells, IL-17 and IL-22 in protecting patients against CMC [29], [43], [112]. Patients with impaired Th17 and Th17-associated cytokines due to mutations in genes such as STAT3 (hyper IgE syndrome, [113], [114]), IL-17RA [115], IL-17F [115], ACT1 [116], STAT1 [117], [118], AIRE [119], [120], IRF8 [121], CARD9 and DECTIN-1 [122], [123], [124] are prone to CMC. In addition, two Mexican patients with a mutation in IL12RB1, suffered from infections with Baccille Calmette-Guérin (BCG) and *C. albicans* and they both died early at ages 4 and 16 [125]. Furthermore, in an international study of 141 patients, 23% of patients lacking IL12RB1were found to suffer from CMC [126]. However, as both IL-12 and IL-23 signal through IL-12Rβ1, this could be due to a lack of responsiveness to either cytokine. IL-23 and IL-6 were detected in vaginal secretions from women infected with vaginal candidiasis [24], [127]. Additionally, healthy PBMCs stimulated with *C. albicans* induced a Th17 response demonstrating that Th17 cells are involved in the defense against *C. albicans* [128]. Furthermore, reduced Th17 responses have been reported in human PBMCs stimulated with *C. albicans* from patients with hyper IgE syndrome, demonstrating that Th17 cells play an important role in the defense against *C. albicans* [113]. These data indicate a protective role for the IL-23/Th17 pathway during *C. albicans* infection, however, heightened IL-17 and IL-23 responses can also negatively regulate Th1 responses to *C. albicans* resulting in increased inflammation and susceptibility to candidiasis [7]. Therefore, similar to IL-12 and IFN-γ, the IL-23/Th17 response needs to be tightly controlled. Th17 cells express IL-10Rα and IL-10 signalling has been shown to control IL-17A producing CD4^+^ T cells in a colitis model [129], suggesting that IL-10 may be an important regulator of IL-23/Th17 responses during anti-fungal immunity.

Studies have demonstrated a role for the IL-23/Th17/IL-17 pathway during *A. fumigatus* infection [7], [130]. *Il12p40^-/-^*, *Il12p35^-/-^* and *Il23p19^-/-^* mice displayed reduced fungal burden in the lungs of *A. fumigatus* infected mice, indicating that a heightened IL-23/Th17 response is associated with susceptibility [7]. However, Dectin-1 and TLR9 deficient mice displayed reduced IL-23, IL-17A and/or IL-22 responses and increased fungal burden in the lungs of *A. fumigatus* challenged mice, indicating that the IL-17 pathway is involved in a protective response to *A. fumigatus* [131], [132]. In contrast, an immunopathogenic role for Dectin-1 and IL-22 has been shown during chronic fungal allergy [104], once again demonstrating the importance for tight regulation of these pathways. IL-17 has been shown to inhibit neutrophil mediated killing of *A. fumigatus* and *in vivo* clearance [7]. Th1 cells, Treg cells, and Th17 cells are all important in producing both an inflammatory and anti-inflammatory response against *A. fumigatus* [7], [48], [133]. The balance between Th17 cells and Treg cells has been suggested to be important in determining whether commensal *C. albicans* causes infection and the same could be true for *Aspergillus* infections [134], [135].

### 3.3 IL-27

The ability of IL-27 to regulate T cell responses has been reviewed elsewhere [136], [137]. Briefly, IL-27 was shown to enhance *in vitro* Th1 cell differentiation [138]. However, more recently, anti-inflammatory properties of IL-27 have been discovered. IL-27 has been shown to inhibit T cell responses by negatively regulating IL-2 signaling and by inducing IL-10 [11], [12], [66], [139], [140], [141], [142]. IL-10 has been shown to inhibit both CD4^+^ and CD8^+^ T cell responses [134], [143]. Additionally, a role in inhibiting Th17 cell differentiation has been demonstrated to protect against Th17 associated disease, such as encephalomyelitis [11], [12], [13], [14]. Th17 cells in encephalomyelitis promotes the development of ectopic lymphoid structures in the central nervous system [144]. Interestingly, IL-27 inhibits the development of ectopic lymphoid structures in inflamed tissues [145]. These functions of IL-27 help protect against T-cell driven pathology. IL-27 has shown contrasting effects on regulatory T cells. It has been shown to promote Treg growth and survival at sites of infection [16] but also to indirectly inhibit Treg development by inhibiting IL-2 production [15], [16].

IL-27 has been reported to be involved in the immune response to various pathogens including *Trypanosoma cruzi* [146], *Leishmania major* [147], [148], [149], *Mycobacteria tuberculosis* [11] and *Toxoplasma gondii* [12], [14], [150]. In addition, a limited number of studies reviewed here, have recently examined the role of IL-27 in response to fungal pathogens*.* The first report in a fungal model demonstrated that *EBI3* levels were significantly induced in human monocyte derived DCs following stimulation with *A. fumigatus* while *IL27* levels only showed a slight increase, demonstrating that *A. fumigatus*-induced IL-27 production is minimal [20]. Indeed, it is possible that the increased *EBI3* levels could actually reflect IL-35 induction rather than IL-27 production, as human monocyte derived tolerogenic DCs express and produce *IL12A* and *EBI3* [151], although this remains to be determined in a fungal model. However, in a later study these authors went on to show that IFN-β treated DCs challenged with *A. fumigatus* resulted in increased *IL27* mRNA levels, IL-12p70 production and increased expression of CD86 and CD83 [152]. While it is well established that IFN-β induces IL-27 [153], this report describes a new role for *A. fumigatus* in IL-27 production [152]. Further, DCs stimulated with IFN-β and challenged with *A. fumigatus* resulted in increased Th1-mediated IFN-γ production [152]. This suggests that IFN-β could be used as a possible therapy to enhance a beneficial Th1 response during infection with *A. fumigatus*.

Type 1 regulatory T cells (Tr1) are instrumental in protecting against Th1/Th17-mediated autoimmunity in mice and reactions to common allergens in humans [154], [155], and may also play an important role during *A. fumigatus* infections [156]. Tr1 cells are Foxp3^-^ regulatory T cells that produce high IL-10 levels and suppress T cell expansion *in vitro* and *in vivo* [157]. Bedke et al. [156] identified Crf-1/p41-specific latent associated peptide^+^ and IL-10^+^ Tr1 cells in healthy humans and in mice following vaccination with an *Aspergillus* peptide (Crf-1/p41) and zymosan. In agreement with previous studies [158], [159], Tr1 cell differentiation *in vivo* was found to be dependent on the aryl hydrocarbon receptor, c-Maf and IL-27. These data indicate that IL-27 plays a role in maintaining anti-fungal immune homeostasis via induction of Tr1 cell differentiation [156].

**Fig. 3:**
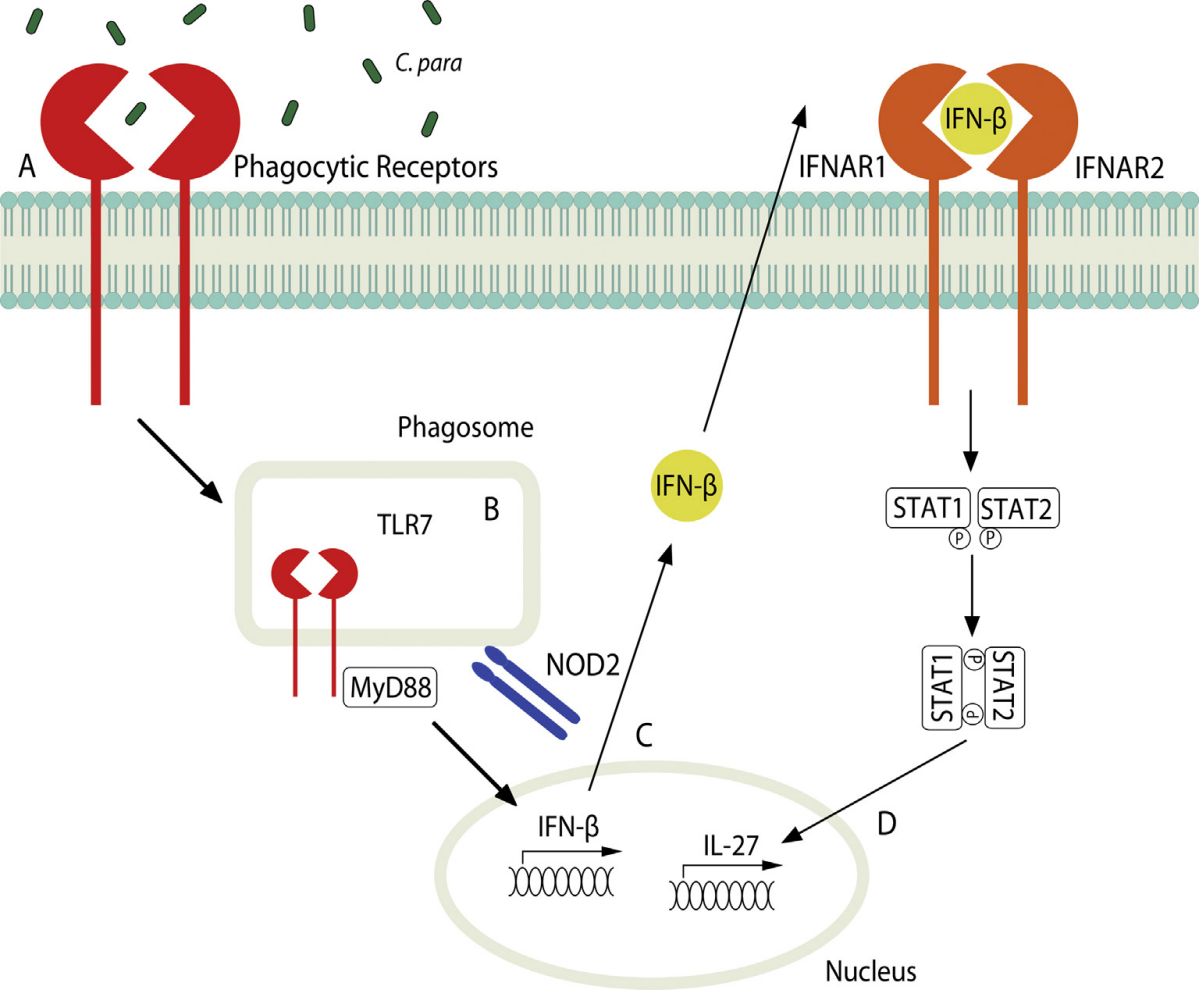
IL-27 induction by *C. parapsilosis*. (A) *C. parapsilosis* engages cell surface receptors on BMDM. (B) *C. parapsilosis* is phagocytosed and TLR7/MyD88 and NOD2 signaling is activated resulting in the induction of nuclear translocation of IFN-β. (C) IFN-β signals through IFNAR1/2-STAT1/2 pathway to (D) induce IL-27 [30].

Several inflammatory cytokines/molecules, such as lipopolysaccharide (LPS) [10], [22], [160], [161], IL-1β [162], Poly(I:C) [161] and IFN-β [152], [153], [163], have been reported to induce IL-27 as a protective response to prevent excess inflammation. Interestingly, increased expression of *IL27* and *EBI3* has been shown in THP-1 cells challenged with heat killed *C. albicans* compared with LPS [22]. In contrast, IL-27 production was reduced in the RAW264.7 macrophage cell line when treated with bakers yeast (saccharomyces cerevisiae)-derived β-glucan (BBG) [160]. In addition, we showed that β-glucan alone does not induce IL-27 in bone marrow derived macrophages (BMDMs) [30]. We also showed that select *Candida* spp. induce IL-27 production, such as *C. parapsilosis, C. glabrata and C. tropicalis* but not *C. albicans*. IL-27 production by *C. parapsilosis* was dependent on phagocytosis, TLR7/MyD88 and nucleotide-binding oligomerisation domain-containing protein (NOD)2 signaling. Further, *C. parapsilosis* induced IFN-β production, which then signaled through the IFN-α/β receptor (IFNAR) and STAT1/2 to induce IL-27 (Fig. 3). Interestingly, while we found that *C. albicans* did not induce IL-27, likely due to the absence of IFN-β induction, it also actively blocked *C. parapsilosis*-induced IL-27 via a soluble mediator [30]. This soluble mediator has yet to be identified, but it is tempting to speculate that BBG might induce the same soluble mediator to block IL-27 production [160]. However, it is interesting to note that live *C. albicans* blocked *C. parapsilosis*-induced IL-27 in mouse BMDMs [30] while heat killed *C. albicans* enhanced LPS-induced IL-27 in the human THP-1 cell line [22]. These discrepancies could be due to differences in mouse vs human IL-27/cells or due to exposure of different ligands on live vs heat killed *C. albicans* and requires further study to unravel these differences.

Similar to infections with other pathogens such as *M. tuberculosis, P. berghei* and *T. gondii* [11], [164], we demonstrated improved clearance of systemic *C. parapsilosis* infection in WSX-1 (*Il27ra*) deficient mice. This was accompanied by increased IFN-γ and IL-17 T cell responses in addition to increased serum pro-inflammatory cytokine levels [30]. While we observed improved fungal clearance and only a modest increase in inflammation, other groups have shown improved pathogen clearance in WSX-1 deficient mice accompanied by fatal organ pathogenesis due to highly elevated pro-inflammatory responses [11], [12], [164]. Interestingly, some studies showed that organ pathology could be prevented by depleting CD4^+^ T cells but not CD8^+^ T cells [12], [164]. As *C. parapsilosis* induced a CD8^+^ T cell biased response rather than a CD4^+^ T cell response, this could account for the improved fungal clearance without inducing fatal organ pathology [30]. These data suggest that blocking IL-27 during infection with *C. parapsilosis* could have therapeutic benefits.

In this review, it has already been mentioned that Th17 cells, IL-17, IL-22 and IL-23 cytokines have important role in protecting patients against CMC [7], [24], [29], [43], [112]. Patients with a gain of function mutation in *STAT1* have higher susceptibility to *Candida* infections [165]. Further, increased *STAT1* function showed increased IL-27, IFN-γ and IFN-α production but reduced IL-17 responses [165], [166], [167]. In a separate study, it has been reported that reduced IFN-γ and IL-27 responses from impaired *STAT1*-expression was observed in two unrelated patients (from Japan and Saudi Arabia) with heterozygous missense mutations in the STAT1 SH2 domain [168]. Further studies in both mice and humans are required to fully unravel the role of IL-27 during anti-fungal immunity.

### 3.4 IL-35

IL-35 is mainly produced by Bregs and Tregs [169]. Stimulation of naïve effector T cells with recombinant IL-35 suppressed T cell proliferation in a murine model and ectopic expression of IL-35 on naïve T cells conferred regulatory activity [71]. IL-35 has been shown to suppress immune responses important for the induction of inflammatory disease in murine models such as collagen-induced arthritis [69], [71]. However, human T regulatory cells do not express IL-35 and as result a role for IL-35 in humans remains unclear [72]. As cytokine subunits are shared between IL and 35, IL-12 and IL-27, mechanistic studies to determine specific roles for IL-35 in the regulation of anti-fungal immunity are challenging.

*Il12p35*^-/-^ mice have been shown to have lower fungal burden during oral candidiasis. As IL-12p35 is a subunit of IL-35, it is possible that IL-35 may play an important role in reducing tissue damage during oral infection [29]. Recently, heat killed *C. albicans* was shown to suppress LPS-induced IL-12p70 production in M2 macrophages due to increased *Ebi3* expression, a subunit of IL-35. Further, the authors demonstrated that *C. albicans* induced IL-35 production, which inhibited an LPS-induced M2 (anti-inflammatory) to M1 (inflammatory) phenotype change in BMDMs, thereby supressing an inflammatory response. These results demonstrate a possible mechanism for how *C. albicans* is able to evade immune detection [23].

## 4 Conclusions

IL-12, IL-23, IL-27 and IL-35 are critical cytokines in innate and adaptive immune responses against fungal infections. They are important in regulating T cell responses and exert their effects on the Th1, Th17 and Treg immune pathways of T cell differentiation. These cytokines are important in regulating the development of inflammation and disease progression. A better understanding of the IL-12 receptor cytokine family, particularly the newer members (IL-27 and IL-35) is invaluable to fully unravel the importance of targeting this family to develop immunotherapies to help fight fungal infections.
